# Components of Golgi-to-vacuole trafficking are required for nitrogen- and TORC1-responsive regulation of the yeast GATA factors

**DOI:** 10.1002/mbo3.168

**Published:** 2014-03-18

**Authors:** Mohammad Fayyadkazan, Jennifer J Tate, Fabienne Vierendeels, Terrance G Cooper, Evelyne Dubois, Isabelle Georis

**Affiliations:** 1 Institut de Recherches Microbiologiques J.-M. Wiame, Laboratoire de Microbiologie, Université Libre de Bruxelles1070, Brussels, Belgium; 2 Laboratoire de Biologie du Transport Membranaire, Institut de Biologie et de Médecine Moléculaires, Université Libre de Bruxelles6041, Gosselies, Belgium; 3 Department of Microbiology, Immunology and Biochemistry, University of Tennessee Health Science CenterMemphis, Tennessee, 38163

**Keywords:** GATA factor, nitrogen availability, rapamycin, vesicular trafficking, yeast

## Abstract

*N*itrogen *c*atabolite *r*epression (NCR) is the regulatory pathway through which *Saccharomyces cerevisiae* responds to the available nitrogen status and selectively utilizes rich nitrogen sources in preference to poor ones. Expression of NCR-sensitive genes is mediated by two transcription activators, Gln3 and Gat1, in response to provision of a poorly used nitrogen source or following treatment with the TORC1 inhibitor, rapamycin. During nitrogen excess, the transcription activators are sequestered in the cytoplasm in a Ure2-dependent fashion. Here, we show that Vps components are required for Gln3 localization and function in response to rapamycin treatment when cells are grown in defined yeast nitrogen base but not in complex yeast peptone dextrose medium. On the other hand, Gat1 function was altered in *vps* mutants in all conditions tested. A significant fraction of Gat1, like Gln3, is associated with light intracellular membranes. Further, our results are consistent with the possibility that Ure2 might function downstream of the Vps components during the control of GATA factor-mediated gene expression. These observations demonstrate distinct media-dependent requirements of vesicular trafficking components for wild-type responses of GATA factor localization and function. As a result, the current model describing participation of Vps system components in events associated with translocation of Gln3 into the nucleus following rapamycin treatment or growth in nitrogen-poor medium requires modification.

## Introduction

Nitrogen is a naturally occurring element that is essential for the growth of all living cells. Many microorganisms have the ability to sense and utilize a broad range of nitrogen sources. When *Saccharomyces cerevisiae* cells are exposed to preferred nitrogen sources (ammonia and glutamine), the expression of genes encoding proteins required for the uptake and utilization of nonpreferred sources (proline, urea, and allantoin) are downregulated. Conversely, when provided with poorly utilized nitrogen sources, expression of these genes is derepressed. The regulatory pathway responsible for this behavior is known as *n*itrogen *c*atabolite *r*epression (NCR) (Cooper 1982; Wiame et al. 1985). Expression of NCR-sensitive genes is mediated by two DNA-binding GATA transcription activators, Gln3 and Gat1/Nil1 (Mitchell and Magasanik 1984; Coffman et al. 1995, 1996; Stanbrough et al. 1995), and is inhibited by the preprion protein Ure2, which acts as a negative regulator of Gln3 and Gat1 (Drillien and Lacroute 1972; Grenson et al. 1974; Courchesne and Magasanik 1988; Blinder et al. 1996; Coffman et al. 1996). In the presence of preferred nitrogen sources, Gln3 and Gat1 are sequestered in the cytoplasm in a Ure2-dependent manner, whereas upon growth in a poor nitrogen supply or upon transferring to nonpreferred nitrogen conditions, the GATA activators relocate to the nucleus and mediate the transcription of NCR-sensitive genes (Cox et al. 2000; Cunningham et al. 2000; Cooper 2002).

The nutrient-responsive TOR complex 1 (TORC1) was also found to participate in coordinating the regulation of GATA factor-mediated expression. Indeed, addition of the immunosuppressant drug rapamycin, inhibiting TORC1, to cells growing in the presence of a good nitrogen source transiently mimics the effects observed with a poor one, that is, nuclear localization and activation of NCR gene expression by Gln3 and Gat1 (Beck and Hall 1999; Cardenas et al. 1999; Hardwick et al. 1999). However, a growing literature demonstrates that multiple distinct regulatory mechanisms are involved in the control of the GATA activators and hence results observed in response to rapamycin treatment cannot be extrapolated to explain responses to nitrogen limitation (Cox et al. 2004; Tate et al. 2005, 2006, 2010; Puria et al. 2008; Georis et al. 2011a; Feller et al. 2013; Rai et al. 2013b; Tate and Cooper 2013). Moreover, although Gln3 and Gat1 are both required for the transcription of most NCR-sensitive genes, the two transcription factors are not always similarly regulated: (1) Gln3 and Gat1 localizations are not similarly sensitive to nitrogen limitation, rapamycin and methionine sulfoximine (Msx, an inhibitor of glutamine synthetase) treatment (Tate et al. 2010; Georis et al. 2011a). (2) In contrast to Gln3, Gat1 nuclear localization is largely Ure2-independent (Georis et al. 2008). (3) Nuclear localization of Gln3 and Gat1 exhibits distinct requirements for the TORC1-regulated phosphatases (Tate et al. 2010; Georis et al. 2011b).

Early investigations of Gln3 regulation established that under steady state, nitrogen-rich conditions, cytoplasmic Gln3 was situated within or tightly associated with a cytoplasmic membrane system (Cox et al. 2002). This membrane association was additionally visualized as Gln3 translocated into and out of the nucleus (Cox et al. 2004). Puria et al. (2008) subsequently confirmed the Gln3 membrane association by demonstrating colocalization of Gln3-Myc^13^ with the Golgi-to-endosome trafficking component Vps10-HA. Endogenous membranes of the protein secretory pathway have also emerged as important platforms for Tor signaling. First, all components of the TORC1 complex (Tor1, Tor2, Kog1, Lst8, and Tco89) (Cardenas and Heitman 1995; Kunz et al. 2000; Chen and Kaiser 2003; Wedaman et al. 2003; Reinke et al. 2004; Aronova et al. 2007) as well as TORC1 regulators (EGO complex) (Dubouloz et al. 2005; Gao and Kaiser 2006; Kim et al. 2008; Binda et al. 2009; Bonfils et al. 2012) and downstream effectors, such as the Tap42–Sit4 phosphatase complex and the AGC kinase Sch9 (Yan et al. 2006; Urban et al. 2007) localize to the late endosome and vacuole membranes. Moreover, the Golgi Ca^2+^/Mn^2+^ATPase Pmr1 has been described to negatively regulate TORC1 function (Devasahayam et al. 2006). Finally, genetic interactions between TORC1 and representative Class C Vps proteins that mediate docking and fusion of vesicles with vacuoles have been described (Aronova et al. 2007; Zurita-Martinez et al. 2007).

Class C and D Vps proteins regulate Golgi-to-vacuole protein transport (Peterson and Emr 2001; Bowers and Stevens 2005): the Class C Vps complex, made up of Vps11/Pep5, Vps18/Pep3, Vps16, and Vps33, is required for docking and fusion at the vacuole (Rieder and Emr 1997; Srivastava et al. 2000; Peterson and Emr 2001; Bowers and Stevens 2005), whereas the Class D proteins (including Vps3, Vps6/Pep12, Vps34 and Vps45) are thought to control vesicular trafficking between the late Golgi and the late endosome (Prescianotto-Baschong and Riezman 2002; Bowers and Stevens 2005). Puria et al. (2008) have previously observed a requirement of Class C and D Vps proteins for Gln3 nuclear translocation after transferring yeast peptone dextrose (YPD) grown cells to proline medium but not after treating them with rapamycin. Aware that Gln3 and Gat1 are sometimes regulated differently, these observations prompted two obvious but important questions: (1) Did Gat1 exhibit the same responses and requirements as Gln3? (2) Would the same responses be observed if a defined, nitrogen-rich rather than complex YPD medium was employed throughout the experiments, thereby eliminating a significant variable from their interpretation?

The first outcome of our study was that Puria et al.'s (2008) observations are medium-specific and hence cannot be generalized. Indeed, although Gln3 nuclear localization is impaired in response to nitrogen limitation but not rapamycin treatment in YPD-pregrown *vps* mutants, we show that in yeast nitrogen base (YNB) ammonia, Vps proteins are required for Gln3 nuclear localization even in response to rapamycin. Therefore, the model describing the requirement of Vps system components for Gln3 trafficking to the nucleus (Puria and Cardenas 2008) requires modification. Second, we show that components of Golgi-to-vacuole trafficking are required for Gat1 function either in YNB-ammonia- or YPD-grown cells treated with rapamycin or transferred to proline medium. Indeed, *vps* mutations reduced the ability of Gat1 to (1) translocate to the nucleus, (2) bind to the Gat1-activated *DAL5* (encoding allantoate permease) promoter, and (3) elicit *DAL5* expression. Additionally, we show that Gat1, like Gln3, fractionates with light intracellular membranes, raising the possibility that its regulation might occur at these locations too. Finally, our observations suggest that Ure2 might function downstream the Vps proteins during GATA factor-mediated signaling.

## Experimental Procedures

### Strains and culture conditions

*S. cerevisiae* strains used in this work are listed in Table 1. Deletion strains involving insertion of kanMX or natMX cassettes were constructed using the short and long flanking homology strategy of Wach (1996) using primers described in Table 1. Gat1-Myc^13^ and Gln3-Myc^13^ protein production was controlled in each *vps* mutant and their levels were comparable to the isogenic wild types (WT). Cultures were grown to midlog phase (A_600nm_ = 0.5–0.55) in YPD medium or YNB minimal medium containing ammonia at a 1% final concentration. Appropriate supplements (100 *μ*g mL^−1^ leucine, 20 *μ*g mL^−1^ uracil, histidine, tryptophan) were added to the medium as necessary to cover auxotrophic requirements. Where indicated, cells were treated for 20 min with 200 ng mL^−1^ rapamycin or transferred to YNB minimal medium containing 0.1% proline for 60 min prior to assay.

**Table 1:** Strains used in this work

Strain	Pertinent genotype	Parent	Complete genotype	Reference	Primer/Reference
TB50	WT		*MATa, leu2-3, 112, ura3-52, trp1, his3, rme1, HMLa*	Beck and Hall (1999)	
TB123	W.T. Gln3-Myc^13^		*MATa, leu2-3, 112, ura3-52, trp1, his4, rme1, HMLa, GLN3-MYC*^*13*^*[KanMX]*	Beck and Hall (1999)	
JK9-3d	W.T.		*MATa, leu2-3, 112, ura3-52, trp1, his4, rme1, HMLa*	Beck and Hall (1999)	
MK23	*vps3*Δ	TB50	*MATa, leu2-3, 112, ura3-52, trp1, his3, rme1, HMLa, vps3::kanMX*	This work	*vps3*: 5′, −42 to −1 and 3′, 3037 to 3076
MK24	*pep5*Δ	TB50	*MATa, leu2-3, 112, ura3-52, trp1, his4, rme1, HMLa, pep5::kanMX*	This work	*pep5*: 5′, −41 to −1 and 3′, 3091 to 3133
MK27	*vps3*Δ Gln3-Myc^13^	FV250	*MATa, leu2-3,112, ura3-52, trp1, his3, rme1, HMLa, vps3::kanMX GLN3-MYC*^*13*^*[HIS3]*	This work	*vps3*: 5′, −42 to −1 and 3′, 3037 to 3076
MK30	*pep5*Δ Gln3-Myc^13^	FV250	*MATa, leu2-3, 112, ura3-52, trp1, his3, rme1, HMLa, pep5::kanMX GLN3-MYC*^*13*^*[HIS3]*	This work	*pep5*: 5′, −41 to −1 and 3′, 3091 to 3133
MK46	*pep3*Δ	TB50	*MATa, leu2-3, 112, ura3-52, trp1, his3, rme1, HMLa, pep3::natMX*	This work	*pep3*: 5′, −42 to −1 and 3′, 2758 to 2801
08047c	*ure2*Δ*vps3*Δ	OK01 X MK23	*MATa, leu2-3, 112, ura3-52, trp1, his4, rme1, HMLa, ure2::natMX, vps3::kanMX*	This work	
OK01	*ure2*Δ	JK9-3d	*MATa, leu2-3, 112, ura3-52, trp1, his4, rme1, HMLa, ure2::natMX*	This work	Ure2-L1, Ure2-L2, Ure2-L3 and Ure2-L4 (Georis et al. 2008)
FV005	*gln3*Δ	TB50	*MATa, leu2-3,112, ura3-52, trp1, his3, rme1, HMLa, gln3::kanMX*	Georis et al. (2008)	
FV006	*gat1*Δ	TB50	*MATa, leu2-3,112, ura3-52, trp1, his3, rme1, HMLa, gat1::kanMX*	Georis et al. (2008)	
FV018	*gat1*Δ Gln3-Myc^13^	TB123	*MATa, leu2-3, 112, ura3-52, trp1, his4, rme1, HMLa, gat1::natMX GLN3-MYC*^*13*^*[KanMX]*	Georis et al. (2008)	
FV063	W.T. Gat1-Myc^13^	TB50	*MATa, leu2-3, 112, ura3-52, trp1, his3, rme1, HMLa, GAT1-MYC*^*13*^*[HIS3]*	Georis et al. (2008)	
FV064	*gln3*Δ Gat1-Myc^13^	FV005	*MATa, leu2-3,112, ura3-52, trp1, his3, rme1, HMLa, gln3::kanMX GAT1-MYC13[HIS3]*	Georis et al. (2008)	
FV250	W.T. Gln3-Myc^13^	TB50	*MATa, leu2-3, 112, ura3-52, trp1, his4, rme1, HMLa, GLN3-MYC*^*13*^*[HIS3]*	Georis et al. (2011b)	
FV390	*vps34*Δ Gln3-Myc^13^	TB123	*MATa, leu2-3, 112, ura3-52, trp1, his3, rme1, HMLa, vps34::natMX, GLN3-MYC*^*13*^*[KanMX]*	This work	*vps34*: 5′, −430 to −407 and −1 to −33; 3′, 2629 to 2664 and 3152 to 3171
FV391	*vps34*Δ	TB50	*MATa, leu2-3,112, ura3-52, trp1, his3, rme1, HMLa, vps34::kanMX*	This work	*vps34*: 5′, −430 to −407 and −1 to −33; 3′, 2629 to 2664 and 3152 to 3171
FV392	*vps34*Δ Gat1-Myc^13^	FV063	*MATa, leu2-3,112, ura3-52, trp1, his3, rme1, HMLa, vps34::kanMX GAT1-MYC*^*13*^*[HIS3]*	This work	*vps34*: 5′, −430 to −407 and −1 to −33; 3′, 2629 to 2664 and 3152 to 3171
FV640	*pep5*Δ Gat1-Myc^13^	FV063	*MATa, leu2-3,112, ura3-52, trp1, his3, rme1, HMLa, pep5::kanMX GAT1-MYC*^*13*^*[HIS3]*	This work	*pep5*: 5′, −41 to −1 and 3′, 3091 to 3133
FV641	*vps3*Δ Gat1-Myc^13^	FV063	*MATa, leu2-3,112, ura3-52, trp1, his3, rme1, HMLa, vps3::kanMX GAT1-MYC*^*13*^*[HIS3]*	This work	*vps3*: 5′, −42 to −1 and 3′, 3037 to 3076
FV642	*vps45*Δ Gat1-Myc^13^	FV063	*MATa, leu2-3,112, ura3-52, trp1, his3, rme1, HMLa, vps45::kanMX GAT1-MYC*^*13*^*[HIS3]*	This work	*vps45*: 5′, −42 to −1 and 3′, 1735 to 1778
FV643	*vps45*Δ	TB50	*MATa, leu2-3,112, ura3-52, trp1, his3, rme1, HMLa, vps45::kanMX*	This work	*vps45*: 5′, −42 to −1 and 3′, 1735 to 1778
FV644	*vps45*Δ Gln3-Myc^13^	FV250	*MATa, leu2-3,112, ura3-52, trp1, his3, rme1, HMLa, vps45::kanMX GLN3-MYC*^*13*^*[HIS3]*	This work	*vps45*: 5′, −42 to −1 and 3′, 1735 to 1778
FV732	*pep3*Δ Gat1-Myc^13^	FV063	*MATa, leu2-3,112, ura3-52, trp1, his3, rme1, HMLa, pep3::kanMX GAT1-MYC*^*13*^*[HIS3]*	This work	*pep3*: 5′, −42 to −1 and 3′, 2758 to 2801
FV733	*pep3*Δ Gln3-Myc^13^	FV250	*MATa, leu2-3,112, ura3-52, trp1, his3, rme1, HMLa, pep3::kanMX GLN3-MYC*^*13*^*[HIS3]*	This work	*pep3*: 5′, −42 to −1 and 3′, 2758 to 2801
FV734	*vps16*Δ Gat1-Myc^13^	FV063	*MATa, leu2-3,112, ura3-52, trp1, his3, rme1, HMLa, vps16::kanMX GAT1-MYC*^*13*^*[HIS3]*	This work	*vps16*: 5′, −42 to −1 and 3′, 2397 to 2439
FV735	*vps16*Δ	TB50	*MATa, leu2-3,112, ura3-52, trp1, his3, rme1, HMLa, vps16::kanMX*	This work	*vps16*: 5′, −42 to −1 and 3′, 2397 to 2439
FV736	*vps16*Δ Gln3-Myc^13^	FV250	*MATa, leu2-3,112, ura3-52, trp1, his3, rme1, HMLa, vps16::kanMX GLN3-MYC*^*13*^*[HIS3]*	This work	*vps16*: 5′, −42 to −1 and 3′, 2397 to 2439

### Quantitative real-time polymerase chain reaction

RNA isolation and cDNA synthesis were conducted as described in (Georis et al. 2009). Quantification of specific cDNA targets was measured by real-time polymerase chain reaction (PCR) performed on a StepOnePlus device (Applied Biosystems, Foster City, CA) using *DAL5* and *GAP1* primers that have been described previously (Georis et al. 2008, 2009). *MEP2*-specific primers are MEP2-O9 (5′-ACGAGGAATCCACTGCTTAC-3′) and MEP2-O10 (5′-TTTCTGCGTCTGTGTTACCC-3′). The values reported represent the averages of at least two experiments from independent cultures; error bars indicate standard errors.

### Chromatin immunoprecipitation

Cell extracts and immunoprecipitations were conducted as described in (Georis et al. 2008, 2009). Concentrations of specific DNA targets in immunoprecipitation (IP) and input (IN) samples were measured by real-time PCR performed on a StepOnePlus device (Applied Biosystems) using primers described in (Georis et al. 2008). The values reported represent the averages of two immunoprecipitations performed in at least two experiments from independent cultures; error bars indicate standard errors.

### Intracellular Gln3 and Gat1 localization

Gln3-Myc^13^ and Gat1-Myc^13^ was visualized by indirect immunofluorescence of whole fixed cells. Immunofluorescence analysis was performed according to standard procedures (Pringle et al. 1991). Cells were fixed for 30 min in formaldehyde (3.7%). They were washed and resuspended in sorbitol buffer (1.2 mol/L sorbitol and 100 mmol/L potassium phosphate, pH 7.5). Cell walls were digested for 50 min at 30°C in sorbitol buffer supplemented with *β*-mercaptoethanol (40 mmol/L final) and lyticase (80 U mL^−1^) (92807 Fluka, St. Gallen, Switzerland). Gln3-Myc^13^ was detected with monoclonal antibody c-Myc (9E10) (Santa Cruz, Dallas, TX) at a working dilution of 1:30. The secondary antibody was Alexa Fluor 488 goat anti-mouse immunoglobulin G (heavy plus light chains) (A-11029; Molecular probes, Carlsbad, CA) (working dilution, 1:200). DNA was stained with DAPI (4′,6′-diamidino-2-phenylindole). Cells were imaged using a fluorescence microscope (Nikon eclipse 80i; Nikon, Tokyo, Japan) equipped with digital sight DS-U3 fluorescence source. Images were captured with a digital camera (Digital sight, DS-U3) and Nikon Instrument Software elements, version 3.22 acquisition software and processed for publication with Photoshop CS (Adobe Systems, San Jose, CA).

To more representatively and completely describe images of Gln3-Myc^13^ and Gat1-Myc^13^ localization appearing in the figures of this paper, primary images were captured and saved in JPEG. We manually scored Gln3-Myc^13^ and Gat1-Myc^13^ localizations in 200 or more cells from six different microscopic fields from which these multiple primary images were taken. For scoring the unchanged images were used. For image publication, a portion of the JPEG files were processed with Photoshop CS (Adobe Systems) where changes were made only to decrease background fluorescence. Cells were classified into one of three categories: cytoplasmic (fluorescence in the cytoplasm only), nuclear–cytoplasmic (fluorescence in the cytoplasm and nucleus), and nuclear (fluorescence in the nucleus only). Although scoring limitations and reproducibility were described in Tate et al. (2006), we emphasize that the nuclear–cytoplasmic category is, of necessity, arbitrary. Placing cells in that category is based on subjective visual evaluation by the individual scoring the cells; it is not an objective instrument-based measurement. When the fluorescent signal is not restricted to a single cellular compartment, scoring depends upon repeated decisions of whether it is nuclear–cytoplasmic or a category flanking it. They will undoubtedly differ in detail from those of another observer, who sets their category dividing lines differently. Our intracellular distributions were scored as consistently as possible. Results were confirmed using independent cultures. Detailed examples of the three scoring categories for Gln3-Myc^13^ and Gat1-Myc^13^ as well as scoring precision (within and between experiments) appear in references (Tate et al. 2006, 2009, 2010; Tate and Cooper 2007, 2008; Georis et al. 2008).

### Subcellular fractionation

Cells were lysed in buffer A containing 50 mmol/L Tris (pH 7.5), 0.2 mol/L sorbitol, 1 mmol/L ethylenediaminetetraacetic acid (EDTA), 1 mmol/L dithiothreitol (DTT) or buffer B containing 0.1 mol/L Tris (pH 7.5), 0.15 mol/L NaCl, 5 mmol/L EDTA. Protease and phosphatase inhibitor cocktails were added for whole-cell extract preparation. Unbroken cells were removed by centrifugation at 500*g* for 10 min. The cell-free extract was centrifuged at 13,000*g* for 15 min to yield P13 (pellet) fraction. The supernatant was then centrifuged at 100,000*g* for 40 min to obtain P100 (pellet) and S100 (supernatant). The different subcellular fractions were analyzed by Western blotting with monoclonal antibodies for c-Myc (Santa Cruz Biotechnology), Pep12 (Invitrogen) and Pgk1 (Invitrogen, Carlsbad, CA). The electrophoresis, blotting, and detection procedures have been described previously (Georis et al. 2009).

## Results

### Class C and D *vps* mutants are defective for activating *DAL5* expression in response to rapamycin or transferring cells from ammonia to proline medium

**Figure 1:**
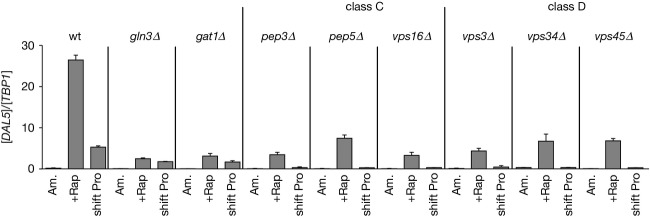
Class C and D Vps proteins requirements for efficient transcription of the Gat1-activated *DAL5* gene following rapamycin treatment or transferring cells from ammonia to proline medium in YNB-grown cells. Total RNA was isolated from WT (TB50), *gln3*Δ (FV005), *gat1*Δ (FV006), the Class C *pep3*Δ (MK46), *pep5*Δ (MK24), and *vps16*Δ (FV735) and the Class D *vps3*Δ (MK23), *vps34*Δ (FV391), and *vps45*Δ (FV643) mutant cells grown in YNB-ammonia medium (Am.) and treated with rapamycin (+Rap) or transferred to proline medium (shift Pro). *DAL5* mRNA levels were quantified by quantitative RT-PCR as described in “Experimental Procedures.” The values reported represent the averages of at least two experiments from independent cultures; error bars indicate standard errors.

In order to examine the requirement of proteins that participate in Golgi-to-vacuole trafficking for the control of NCR-sensitive gene expression, we assayed *DAL5* expression in YNB-ammonia-grown untreated, rapamycin-treated or proline-transferred WT, *gln3*Δ and *gat1*Δ as well as Class C (*pep3*Δ, *pep5*Δ and *vps16*Δ) and D (*vps3*Δ, *vps34*Δ and *vps45*Δ) *vps* mutant cells (Fig. 1). The expression levels exhibited by rapamycin-treated or proline-transferred WT cells were clearly impaired in *gln3*Δ or *gat1*Δ-mutant cells, demonstrating that *DAL5* transcription requires the simultaneous presence of Gln3 and Gat1 when YNB-ammonia-grown cells are transferred to proline medium, or treated with rapamycin (Georis et al. 2008). After a transfer to proline, *DAL5* expression was lost in all *vps* mutant cells compared with a WT strain, whereas residual expression still occurred after rapamycin treatment (Fig. 1). Impaired *DAL5* expression in rapamycin-treated or *vps* mutant cells transferred to proline could be attributed to loss of Gln3 function alone, Gat1 alone or the functions of both Gln3 and Gat1 together. Importantly, *DAL5* expression decreased more when *vps* mutants were transferred from ammonia to proline medium than in either the *gln3*Δ or *gat1*Δ strains, suggesting that components of the Golgi-to-vacuole trafficking system may be required for both Gat1 and Gln3 function.

### Components of Golgi-to-vacuole trafficking are required for efficient GATA factor binding to the Gat1-activated *DAL5* promoter

**Figure 2:**
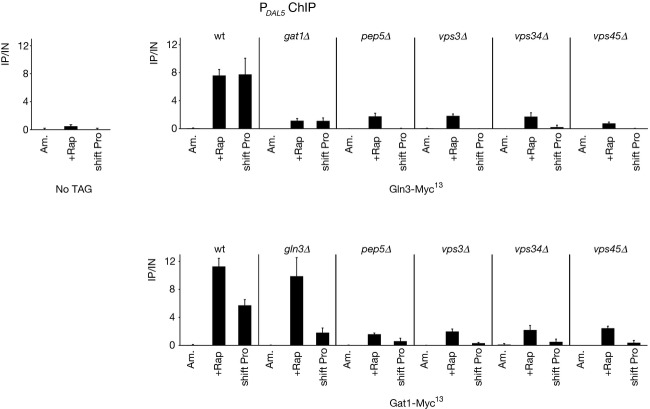
Gln3-Myc^13^ and Gat1-Myc^13^ binding to the *DAL5* promoter in WT, *gln3*Δ and *gat1*Δ cells as well as in the Class C *pep5*Δ mutant and the Class D *vps3*Δ, *vps34*Δ, and *vps45*Δ mutant cells in response to rapamycin treatment or transferring cells from YNB-ammonia to YNB-proline medium. Untagged WT (TB50), *GLN3*-*MYC*^*13*^ WT (FV250), *GLN3*-*MYC*^*13*^ *gat1*Δ (FV018), *GLN3*-*MYC*^*13*^ *pep5*Δ (MK30), *GLN3*-*MYC*^*13*^ *vps3*Δ (MK27), *GLN3*-*MYC*^*13*^ *vps34*Δ (FV390), *GLN3*-*MYC*^*13*^ *vps45*Δ (FV644), *GAT1*-*MYC*^*13*^ WT (FV063), *GAT1*-*MYC*^*13*^ *gln3*Δ (FV064), *GAT1*-*MYC*^*13*^ *pep5*Δ (FV640), *GAT1*-*MYC*^*13*^ *vps3*Δ (FV641), *GAT1*-*MYC*^*13*^ *vps34*Δ (FV392), and *GAT1*-*MYC*^*13*^ *vps45*Δ (FV642) cells were grown in YNB-ammonia medium (Am.) and treated with rapamycin (+Rap) or transferred to proline medium (shift Pro). ChIP was performed using antibodies against c-*myc* as described in “Experimental Procedures.” qPCR of IP and IN fractions was performed with primers specific for the *DAL5* promoter (DAL5P) and for a region 2.5 kb upstream of the *DAL5* open reading frame as a control (DAL5U). For each immunoprecipitation, IP/IN values were calculated as follows: ([DAL5P]^IP^/[DAL5P]^IN^ − [DAL5U]^IP^/[DAL5U]^IN^). The values reported represent the averages of two immunoprecipitations performed in at least two experiments from independent cultures; error bars indicate standard errors.

To test whether the observed requirement of representative Class C and D Vps proteins for *DAL5* expression following rapamycin treatment or transfer to proline medium affected GATA factor binding to the *DAL5* promoter_,_ chromatin immunoprecipitation experiments were performed in the strains described above. Gat1-dependent Gln3-Myc^13^ binding to the *DAL5* promoter was elicited in rapamycin-treated or proline-transferred WT cells (Fig. 2) (Georis et al. 2008). Cells lacking Gat1 or any of the Vps proteins tested displayed an impaired Gln3-Myc^13^ binding in both rapamycin-treated and proline-transferred cultures (Fig. 2). However, some residual binding did remain in the rapamycin-treated cells. Gat1-Myc^13^ was efficiently recruited to the *DAL5* promoter in rapamycin-treated WT cells (Georis et al. 2008), but only about half as efficiently following transfer to proline medium (Fig. 2). Gat1 binding in cells transferred to proline medium required Gln3 and all of the Vps components tested. In contrast, high level Gat1-Myc^13^ binding to the *DAL5* promoter following rapamycin treatment required only the Vps proteins assayed but not Gln3. This observation demonstrates that Gat1 binding in rapamycin-treated cells largely requires functional Golgi-to-vacuole trafficking components, independent of Gln3 function. On the other hand, impaired Gln3 binding observed in rapamycin-treated or proline-transferred *vps* mutants, could be either due to a direct requirement of the Golgi-to-vacuole trafficking component proteins we assayed or as a consequence of impaired Gat1 binding.

### Efficient nuclear GATA factor localization in rapamycin-treated or proline-transferred cells requires Class C and D Vps proteins

**Figure 3:**
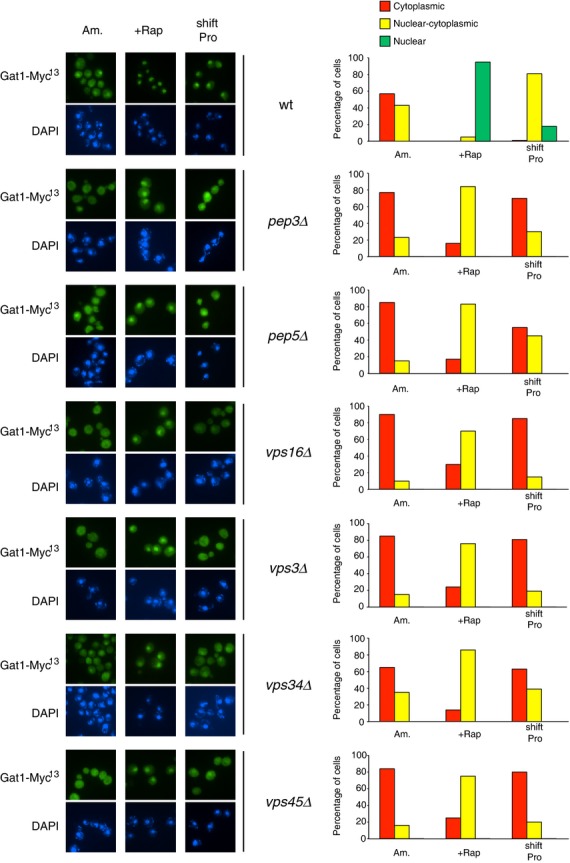
Requirements of Class C and D Vps proteins for intracellular Gat1-Myc^13^ localization in response to rapamycin or transferring cells from YNB-ammonia to YNB-proline medium. *GAT1-MYC*^*13*^ WT (FV063), Class C *pep3*Δ (FV732), *pep5*Δ (FV640), and *vps16*Δ (FV734) and Class D *vps3*Δ (FV641), *vps34*Δ (FV392) ,and *vps45*Δ (FV642) mutant cells were grown in YNB-ammonia medium (Am.). Cells were treated with rapamycin (+Rap) or transferred to proline medium (shift Pro). The cultures were sampled for indirect immunofluorescence assay of Gat1-Myc^13^ localization. Indirect immunofluorescence assays were performed and imaged as described in Experimental Procedures. Images from which the histograms were derived are displayed on the left hand side of the figure. The upper member of each pair depicts green Gat1-Myc^13^-derived fluorescence and the lower one shows DAPI-positive material fluorescence. For each histogram, displayed at the right hand side of the figure, cells were scored for intracellular Gat1-Myc^13^ localization (cytoplasmic red bars, nuclear–cytoplasmic, yellow bars; nuclear, green bars) using criteria described in Experimental Procedures. When no histogram bar is visible on the graph, that is because there were no cells found in scoring category considered. The values reported represent the averages of at least two experiments from independent cultures.

**Figure 4:**
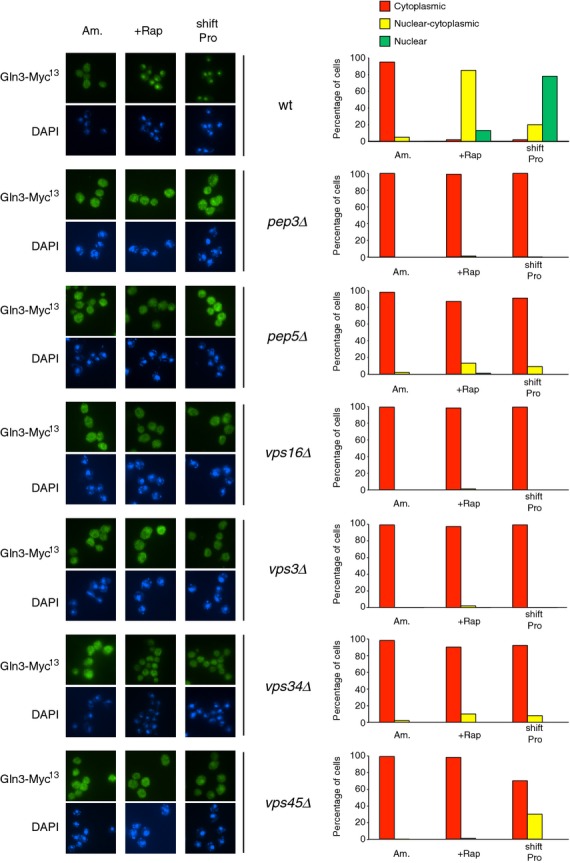
Requirements of Class C and D Vps proteins for intracellular Gln3-Myc^13^ localization in response to rapamycin or transferring cells from YNB-ammonia to YNB-proline medium. *GLN3-MYC*^*13*^ WT (FV250), Class C *pep3*Δ (FV733), *pep5*Δ (MK30), and *vps16*Δ (FV736) and Class D *vps3*Δ (MK27), *vps34*Δ (FV390), and *vps45*Δ (FV644) mutant cells were grown in YNB-ammonia medium (Am.) and treated with rapamycin (+Rap) or transferred to proline medium (shift Pro). The experimental format and data presentation are the same as those in Figure 3.

To determine whether impaired GATA factor binding to DNA derived from impaired nuclear localization in the *vps* mutants, we characterized Gat1-Myc^13^ and Gln3-Myc^13^ localization using indirect immunofluorescence. Gat1-Myc^13^ nuclear localization in rapamycin-treated or proline-transferred cells pregrown in YNB-ammonia was reduced in the Class C and D *vps* mutants relative to WT (Fig. 3). Following rapamycin treatment, Gat1-Myc^13^ was mainly nuclear–cytoplasmic in all tested *vps* mutant cells rather than fully nuclear as occurred in WT cells. In cells transferred to proline medium, the fraction of *pep3*Δ, *pep5*Δ, *vps16*Δ, *vps3*Δ, *vps34*Δ, *vps45*Δ cells where Gat1-Myc^13^ was nuclear or nuclear–cytoplasmic also decreased, accompanied by increased cytoplasmic Gat1-Myc^13^ localization. In none of the cases, however, were the Vps protein requirements absolute. These observations indicate that Class C and D Vps proteins are partially required for efficient Gat1 translocation to the nucleus. In contrast, Class C and D *vps* mutations totally abolished Gln3-Myc^13^ nuclear localization not only in cells transferred to proline medium but also, surprisingly, after rapamycin treatment (Fig. 4). These observations, obtained with YNB medium, contrast with a previous report showing that Golgi-to-vacuole trafficking components were dispensable for rapamycin-elicited Gln3 nuclear translocation (Puria et al. 2008).

### A media-dependent role of Class C and D Vps proteins for Gln3 and Gat1 nuclear translocation and *DAL5* gene activation

**Figure 5:**
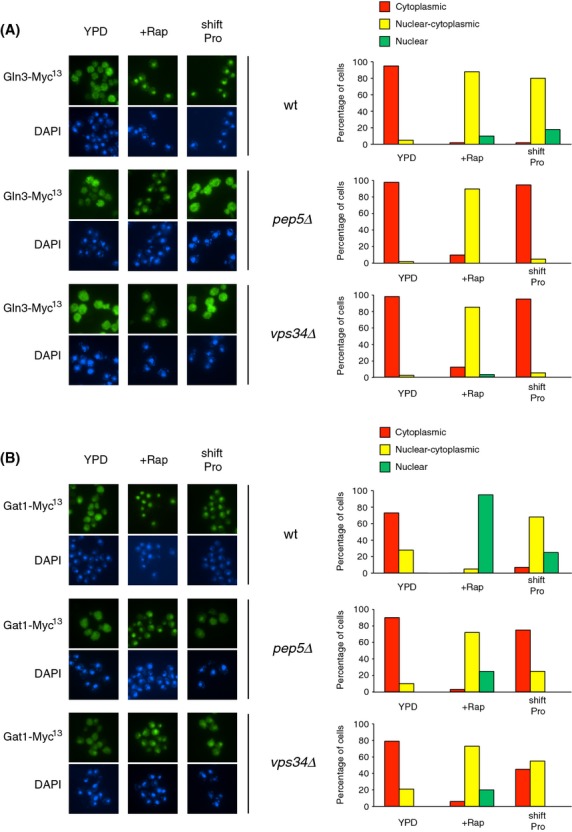
Requirements of Class C and D Vps proteins for intracellular Gln3-Myc^13^ and Gat1-Myc^13^ localization in YPD growth conditions. *GLN3*-*MYC*^*13*^ WT (FV250), *pep5*Δ (MK30), *vps34*Δ (FV390), *GAT1*-*MYC*^*13*^ WT (FV063), *pep5*Δ (FV640), and *vps34*Δ (FV392) cells were grown in YPD medium and treated with rapamycin (+Rap) or transferred to proline medium (shift Pro). The experimental format and data presentation are the same as those in Figure 3 with one exception that all the strains were grown in YPD medium instead of YNB-ammonia.

To test if the apparent discrepancy between present and previously reported results was due to a technical problem or the different growth conditions employed, we characterized Gln3-Myc^13^ localization in WT, *pep5*Δ (chosen as Class C representative) and *vps34*Δ (chosen as Class D representative) mutant cells using the same growth conditions as Puria et al. (2008), that is, in YPD medium (untreated, rapamycin-treated or after transfer to proline medium). Our observations (Fig. 5A) were in agreement with previous studies showing that mutations in Class C and D *VPS* genes impair Gln3 nuclear localization following a transfer to proline medium but not to rapamycin treatment (Puria et al. 2008), indicating that the observed discrepancy was indeed due to the nature of the growth medium.

The same procedure was followed to assess Gat1-Myc^13^ localization. As occurred in YNB-ammonia-grown WT cells (Fig. 3), rapamycin addition to YPD-grown cells led to a fully nuclear localization of Gat1-Myc^13^ (Fig. 5B). Deletion of *VPS34* or *PEP5* in rapamycin-treated cells reduced Gat1-Myc^13^ nuclear localization by increasing the fraction of cells in which Gat1-Myc^13^ was nuclear–cytoplasmic at the expense of cells where Gat1-Myc^13^ was fully nuclear. Gat1-Myc^13^ nuclear localization in response to transferring cells to proline medium was also partially impaired in the *vps34*Δ and *pep5*Δ mutants relative to WT. This was characterized by an increase in the fraction of cells where Gat1-Myc^13^ was cytoplasmic and a decrease in the fraction of cells where Gat1-Myc^13^ was nuclear and/or nuclear–cytoplasmic.

**Figure 6:**
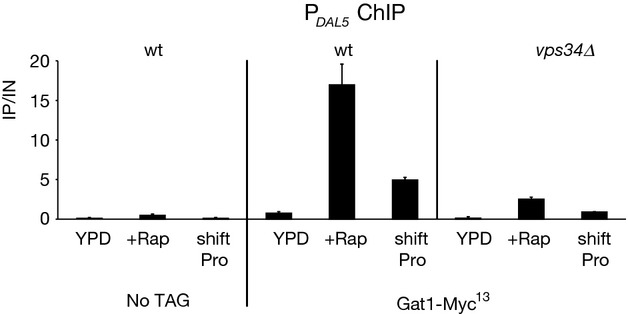
Gat1-Myc^13^ binding to the *DAL5* promoter in WT and *vps34*Δ strains in response to rapamycin or transfer of YPD-grown cells to YNB-proline medium. Untagged WT (TB50), *GAT1*-*MYC*^*13*^ WT (FV063) and *GAT1*-*MYC*^*13*^ *vps34*Δ (FV392) cells were grown in YPD medium and treated with rapamycin (+Rap) or transferred to proline medium (shift Pro). ChIP was performed as described in Figure 2.

To test whether the observed requirement of Class C and D Vps proteins for Gat1-Myc^13^ nuclear localization upon rapamycin treatment or transferring cells to proline medium paralleled its ability to bind to the *DAL5* promoter, chromatin immunoprecipitation experiments were performed in WT and *vps34*Δ cells (Fig. 6). Much more Gat1-Myc^13^ was recruited to the *DAL5* promoter in rapamycin-treated WT cells compared with untreated YPD-grown cells. In contrast, Gat1-Myc^13^ binding to the *DAL5* promoter in *vps34*Δ cells treated with rapamycin or transferred to proline medium was reduced.

**Figure 7:**
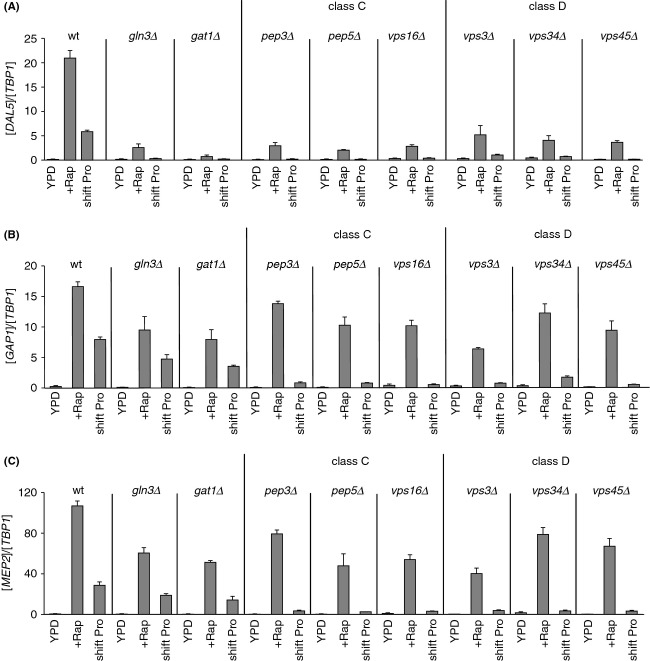
Class C and D Vps protein requirements for transcription of the GATA factor-activated genes *DAL5, GAP1* and *MEP2* in response to rapamycin or transfer of YPD-grown cells to YNB-proline medium. The strains, experimental format and data presentation are the same as those in Figure 1 with one exception that all the strains were grown in YPD medium instead of YNB-ammonia. (A) *DAL5*. (B) *GAP1*. (C) *MEP2*.

Finally, we determined the transcription profiles of *DAL5, GAP1,* and *MEP2* genes in YPD-grown, untreated, rapamycin-treated, or proline-transferred WT, *gln3*Δ, *gat1*Δ and Class C and D *vps* mutant cells (Fig. 7). The *DAL5* expression levels elicited by rapamycin-treated or proline-transferred WT cells were clearly decreased in *gln3*Δ or *gat1*Δ mutant cells, demonstrating that, as occurred in YNB medium, *DAL5* transcription requires both GATA factors under these conditions (Fig. 7A). After rapamycin treatment or transfer to proline medium, *DAL5* expression was clearly reduced in all *vps* mutants relative to WT cells (Fig. 7A). As Gln3-Myc^13^ nuclear localization is largely intact, impaired *DAL5* expression in rapamycin-treated *vps* mutant cells should result from the reduced Gat1-Myc^13^ nuclear localization and DNA binding. On the other hand, the impaired *DAL5* expression exhibited by the *vps* mutants in response to transferring cells into proline medium could be attributed to the impaired nuclear localization of either Gln3-Myc^13^, Gat1-Myc^13^ or of both GATA activators.

*GAP1* or *MEP2* expression levels in YPD-grown WT cells treated with rapamycin or transferred to proline medium were only slightly reduced in *gln3*Δ or *gat1*Δ mutant cells, demonstrating that either Gln3 or Gat1 alone could sustain *GAP1* or *MEP2* expression in rapamycin-treated cells or in cells transferred to proline medium (Fig. 7B and C). Consistent with previous data (Puria et al. 2008), only in cells transferred to proline medium but not those treated with rapamycin, were *GAP1* and *MEP2* expression levels reduced in all *vps* mutant cells compared with a WT strain (Fig. 7B and C). The unaffected expression observed after rapamycin treatment in the *vps* mutants correlated with the intact nuclear translocation of Gln3. On the other hand, impaired expression observed in *vps* mutant cells transferred to proline medium was in agreement with the impaired nuclear translocation of both Gln3 and Gat1.

### A fraction of Gat1 associates with light intracellular membranes

**Figure 8:**
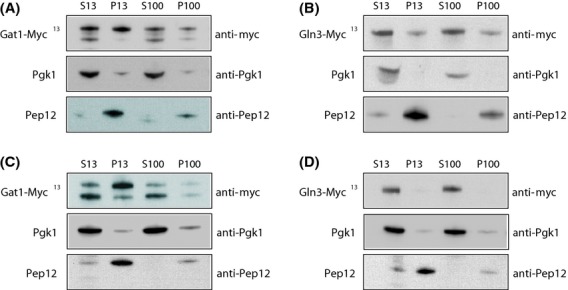
Subcellular fractionation of Gat1-Myc^13^ and Gln3-Myc^13^. Cell-free lysates from YPD-grown *GAT1*-*MYC*^*13*^ WT (FV063; A and C) and *GLN3*-*MYC*^*13*^ WT (FV250; B and D) cells were subjected to differential centrifugation to yield low-speed pellet (P13), supernatant (S13), high-speed pellet (P100), and soluble (S100) fractions. Equal cell equivalents were examined by Western blot to detect Gln3-Myc^13^, Gat1-Myc^13^, Pep12, and Pgk1. Protein extracts were prepared using a lysis buffer lacking NaCl (A and B) or containing 0.15 mol/L NaCl (C and D).

Several regulators of GATA factor-activated genes, for example, TORC1, Tap42-Sit4 phosphatase, as well as Gln3 have been shown to be associated with intracellular light membranes (Cardenas and Heitman 1995; Kunz et al. 2000; Chen and Kaiser 2003; Wedaman et al. 2003; Reinke et al. 2004; Yan et al. 2006; Aronova et al. 2007; Puria et al. 2008). These findings, and our observations that *vps* mutant cells exhibit defects in Gat1 localization and function, raised the possibility that it too might be associated with light membranes. Therefore, Gat1-Myc^13^ subcellular fractionation was performed to assess this possibility (Fig. 8A). To evaluate the degree to which our procedure cleanly separated soluble from insoluble proteins, we examined the distribution of the cytosolic marker Pgk1 and the membrane-associated protein, Pep12. As expected, Pgk1 was recovered primarily in the supernatant fraction, whereas the endosomal marker Pep12 was distributed between both the P13 and P100 fractions (Fig. 8A), as described previously (Becherer et al. 1996). Gat1-Myc^13^ was detected as a typical double band, corresponding to two isoforms possessing different N-termini (Rai et al. 2013a). In WT cells, Gat1-Myc^13^, like Gln3-Myc^13^, was fractionated not only with heavy (P13) membranes (Fig. 8A for Gat1-Myc^13^ and B for Gln3-Myc^13^) known to contain plasma and endoplasmic reticulum membranes but also with lighter (P100) membranes containing Golgi and the endosomal marker Pep12. In contrast with membrane-associated Pep12, significant portions of Gat1-Myc^13^ and Gln3-Myc^13^ were still detected in the S13 and S100 fractions along with the cytosolic marker Pgk1, indicating that only a portion of Gat1-Myc^13^ and Gln3-Myc^13^ were associated with intracellular membranes.

Gln3 association with intracellular membranes has already been reported as likely being peripheral as indicated by its sensitivity to extraction with NaCl (Puria et al. 2008). To test this characteristic for Gat1, protein extracts were prepared using a stringent lysis buffer (containing 0.15 mol/L NaCl). Under such stringent conditions, Gat1-Myc^13^ fractionated largely with heavy (P13) membranes (Fig. 8C) but only residually with lighter (P100) ones. Interestingly, the two isoforms of Gat1-Myc^13^ were unevenly distributed between the supernatant and the pellet fractions. The faster migrating isoform, beginning at Gat1 methionines 95 or 102 (Rai et al. 2013a), fractionated mainly in the soluble fractions, whereas the slower migrating isoform, beginning at Gat1 methionine 40 (Rai et al. 2013a), was mainly associated with the P13 fraction. As expected, Gln3-Myc^13^ was not detected in the pellet fraction under these stringent conditions (Fig. 8D). These findings indicate that Gat1 appears to be more stably associated with light membranes than Gln3.

### Ure2 seems to act downstream of Vps3 to control *DAL5* expression

Ure2 is a well-known negative regulator of GATA factor-mediated gene expression (Grenson et al. 1974; Courchesne and Magasanik 1988). Aiming at determining the epistatic relation between the *ure2*Δ and *vps* mutations, all attempts to delete *URE2* in *vps34*Δ, *pep5*Δ, or *vps45*Δ mutant cells failed, suggesting that combining the *ure2*Δ with either *vps34*Δ, *pep5*Δ, or *vps45*Δ mutations might be resulting in synthetic lethality. To test this possibility, tetrad analysis was conducted in crosses between the *vps34*Δ, *pep5*Δ, and *vps45*Δ and *ure2*Δ strains. Haploid meiotic progeny generated by sporulation of heterozygous diploids containing the above mutations carried only one mutation. No meiotic segregants carrying a double mutation were recovered, confirming that these double mutants were synthetically lethal. Double *ure2*Δ*vps3*Δ mutants, however, were recovered on YPD medium following sporulation of a *VPS3ure2*Δ/*vps3*Δ*URE2* heterozygous diploid strain, but these were unable to grow in YNB-ammonia medium.

**Figure 9:**
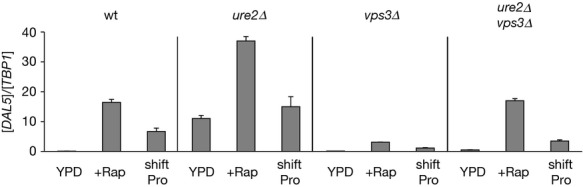
Epistatic Relation between Ure2 and Vps3. WT (TB50), *ure2*Δ (OK01), *vps3*Δ (MK23), *ure2*Δ*vps3*Δ (08047c) cells were grown in YPD medium and treated with rapamycin (+Rap) or transferred to proline medium (shift Pro). *DAL5* mRNA levels were quantified by quantitative RT-PCR as described in “Experimental Procedures.” The experimental format and data presentation are the same as those in Figure 1.

*DAL5* expression was assayed in YPD-grown untreated, rapamycin-treated, or proline-transferred WT, *ure2*Δ, *vps3*Δ, and *ure2*Δ*vps3*Δ cells (Fig. 9). In YPD-grown cells, high-level *DAL5* expression observed in a *ure2*Δ was lost upon additionally deleting *VPS3*. However, in response to rapamycin treatment or a transfer to proline, the elevated *DAL5* expression levels observed in *ure2*Δ were only lowered, but not lost in the double mutant. In fact, the double mutant exhibited a phenotype resembling the WT. Altogether, these observations suggest that the *vps3*Δ and *ure2*Δ mutations more likely only compensate the effects of one another.

## Discussion

In this report, we show that mutations in Class C and D Vps proteins led to defects in *DAL5* expression, after treating cells with rapamycin or transferring them to YNB-proline medium. These defects correlated with altered Gat1 and Gln3 nuclear localization and DNA binding. Vps protein requirements for Gat1 localization and function were observed in cells grown either in defined, nitrogen-rich or complex YPD medium, whereas the requirements of representative Vps proteins for Gln3 function were media-specific: a requirement after rapamycin treatment was observed in YNB and not YPD medium. The results in YPD, but not YNB medium were in agreement with Puria et al.'s (2008) initial conclusions. However, the media-dependence of the Vps proteins requirements for the rapamycin control of Gln3 localization and function suggests that the previously published model describing Vps protein participation in GATA factor regulation may not be correct and hence in need of revision (Puria and Cardenas 2008).

The influence of medium composition (YPD vs. YNB) on the Vps requirement for the control of Gln3 localization may not be too surprising. A previous report has indicated that key components of the medium, such as zinc or the pH of the medium, can influence Gln3 cellular localization and GATA factor responses (Feller et al. 2006). Several differences exist between YPD and YNB media, including the pH and the quality of the nitrogen sources provided. Our YNB cultures started at a pH of 5 and were harvested at a pH of 2.7, whereas in YPD, starting and ending pHs were 6 and 4.8, respectively. Another major difference between the YPD and YNB-ammonia media is the quality of the nitrogen source, influencing the yeasts' growth rate (faster on YPD than on YNB). A third major difference is the availability of all amino acids in YPD medium, whereas YNB-ammonia-grown cells need to synthesize their own amino acids (Magasanik and Kaiser 2002; Ljungdahl and Daignan-Fornier 2012). Although it is still not obvious if and how the Vps proteins may be connected to the presence of external amino acids, we speculate that our data do not exclude the possibility that Vps proteins could be required for Gln3 relocation to the nucleus at multiple steps other than only those associated with protein trafficking to the vacuole. In mammalian cells, it has been reported that Vps34 is required for amino acids sensing and transmitting the stimulatory effects of amino acids to the TOR pathway (Byfield et al. 2005; Nobukuni et al. 2005; Backer 2008). Moreover, and in agreement with their multiple functions, two distinct Vps34 complexes function in autophagy and carboxypeptidase Y sorting in response to nitrogen starvation (Kihara et al. 2001).

In addition to the requirement of normal Golgi-to-vacuole trafficking components for Gln3 and Gat1 nuclear localization, we show that Gat1, like Gln3 (Puria et al. 2008), associates with light membranes probably derived from the Golgi apparatus, although the former seems to be more stably associated with light membranes than the latter. Interestingly, the two Gat1 isoforms appeared to behave differently: irrespective of the lysis conditions, the faster migrating form was always observed in the cytosolic fraction, whereas the slower migrating form was more associated with the membrane fraction. It is possible that the 55 N-terminal amino acids lacking in the shorter isoform (Rai et al. 2013a) are determinants of interactions leading to membrane association. In line with this hypothesis, mutants affecting the N-terminal methionines display altered cytoplasmic retention in repressing conditions although this did not affect Gat1's transactivation capacities (Rai et al. 2013a).

Our results also suggest that Ure2 most likely participates in GATA factor regulation downstream the Vps proteins. Aware that genetic interactions exist between *URE2* and several *VPS* genes (Costanzo et al. 2010; Hoppins et al. 2011), the synthetic lethality exhibited upon deleting *URE2* in combination with *VPS34, VPS45,* or *PEP5* was not very surprising. The *ure2*Δ*vps3*Δ double mutant exhibited a WT transcription profile under derepressive conditions, thus, suggesting that Vps3 may be dispensable for GATA factor function, at least when Ure2 is absent. Multiple speculative explanations are possible. Among them, Vps components could be incorporated in a membrane-based nitrogen sensing system where mutating the Vps components would impair transmission of the signal and thus leading to constitutive negative regulation of the GATA factors in a Ure2-dependent manner. However, adding a *ure2* mutation to a *vps* mutation (i.e., *ure2*Δ*vps3*Δ double mutant) will relieve the negative regulation of the GATA factors. Accordingly, Ure2 would appear to function downstream of the VPS system. Another possibility might be that Vps proteins and Ure2 might exert GATA factor regulation through separate pathways. Taking advantage of the inability of the *ure2*Δ*vps3*Δ double mutant to grow on YNB-ammonia medium, it could prove useful to select suppressors or mutants able to bypass this lethality. This would enable the identification of new components involved either in GATA factor regulatory pathways or in membrane trafficking as well as other potential unrelated roles of Ure2.

In *S. cerevisiae*, some, but not all, amino acids are compartmentalized in the vacuoles (Wiemken and Durr 1974; Kitamoto et al. 1988; Sekito et al. 2008). Major known regulators of GATA factor-mediated gene activation (TORC1, Tap42-Sit4 and Gln3) have been localized to membranes of the secretory pathway (Cardenas and Heitman 1995; Kunz et al. 2000; Chen and Kaiser 2003; Wedaman et al. 2003; Reinke et al. 2004; Yan et al. 2006; Aronova et al. 2007; Puria et al. 2008). Moreover, the EGO complex, which is thought to couple amino acid signals to TORC1 activity, resides on the vacuolar membrane (Binda et al. 2009). Together, our results and these observations are consistent with the conclusion that Class C and D Vps proteins are required for the responses of GATA factor intracellular localization and function to changing nitrogen availability. Further, the data suggest that cytoplasmic membranes, which are associated with Golgi-to-vacuolar trafficking, appear to be additionally involved with the generation and/or implementation of these responses.

Together with our data, a recent report (Han and Emr 2011) supports the growing view that transcription factor regulation occurs not only inside the nucleus, but potentially at cytoplasmic compartments as well. In this manner, cytoplasmic signals may directly or indirectly regulate the function of transcription factors, thus strengthening the connection between gene expression and the intra- and extracellular environments to which it responds.
